# Vitreoretinal instruments: vitrectomy cutters, endoillumination and wide-angle viewing systems

**DOI:** 10.1186/s40942-016-0052-9

**Published:** 2016-12-05

**Authors:** Paulo Ricardo Chaves de Oliveira, Alan Richard Berger, David Robert Chow

**Affiliations:** 1 Toronto Retina Institute, 208-6 Maginn Mews, North York, ON M3C 0G9 Canada; 2 Department of Ophthalmology and Vision Sciences, St. Michael’s Hospital, University of Toronto, Toronto, ON Canada

**Keywords:** Endoillumination, Vitrectomy cutters, Wide-angle viewing systems

## Abstract

There have been many advances in vitreoretinal surgery since Machemer introduced the concept of pars plana vitrectomy, in 1971. Of particular interest are the changes in the vitrectomy cutters, their fluidics interaction, the wide-angle viewing systems and the evolution of endoillumination through the past decade and notably in the last few years. The indications of 27-gauge surgery have expanded, including more complex cases. Cut rates of up to 16,000 cuts per minute are already available. New probe designs and pump technology have allowed duty cycle performances of near 100% and improved flow control. The smaller vitrectomy diameter can be positioned between narrow spaces, allowing membrane dissection and serving as a multifunctional instrument. Enhanced endoillumination safety can be achieved by changing the light source, adding light filters, increasing the working distance and understanding the potential interactions between light and vital dyes commonly used to stain the retina. Wide-angle viewing systems (contact, non-contact or a combination of both) provide a panoramic view of the retina. Non-contact systems are assistant-independent, while contact systems may be associated with better image resolution. This review will cover some current aspects on vitrectomy procedures, mainly assessing vitrectomy cutters, as well as the importance of endoillumination and the use of wide-angle viewing systems.

**Electronic supplementary material:**

The online version of this article (doi:10.1186/s40942-016-0052-9) contains supplementary material, which is available to authorized users.

## Background

Over 45 years ago, the idea of removing vitreous through a smaller aperture, with minimum trauma to the anterior compartment, was inspiring and revolutionary. In 1971, Machemer et al. introduced the concept of pars plana vitrectomy. The first vitreous cutter consisted of a micromotor which activated a drill bit inside a hypodermic needle, adapted on a plastic syringe and powered by a regular battery [1, 2]. The next significant step was taken by Conor O’Malley and Ralph Hein, which developed the three-port vitrectomy with a 20-gauge system as well as a lightweight, reusable, bellows-driven, pneumatic, axial cutter driven by the Ocutome 800 console (Berkley Bioengineering, 1972) [3]. Since those humble beginnings there have been innumerable advances in vitrectomy surgery. Significant improvements have occurred not only with vitrectomy probes, which go faster and are smaller, but also in countless other aspects of our surgical environment including fluidics, endoillumination, handheld instrumentation, wound construction, console design and viewing systems, among others. As technology continues to improve it is important for surgeons to understand the implications that each of these innovations will have on their surgical performance and also how these variables will interact with each other. This article will review some advances in vitrectomy technology focusing on vitreous cutters, endoillumination and wide-angle viewing systems.

## Vitrectomy cutters

Since the 1970s, vitreous cutters have been modified to achieve high performance surgeries while maintaining safety. Numerous components such as the cutter size, cutting speed, port geometry, blade design and duty cycle, can alter the surgical efficiency and impact the postoperative results.

### Cutter size

One of the major advances in retinal surgery is the reduced size of cutters. Smaller vitrectomy probes allowed the transition to the microincision vitrectomy system (MIVS), introduced in 2002 by Fujii et al., using 25-gauge instruments and followed by Eckardt in 2005, with 23-gauge cutters [4–6]. Subsequent years have shown that the modern MIVS have numerous advantages over 20-gauge vitrectomy and are associated with better patient comfort, less conjunctival scarring, less postoperative inflammation and earlier visual recovery [7–11]. More recently, 27-gauge instruments released by different companies have shown encouraging results [7–9, 12, 13].

In 2010, the first series of cases using 27-gauge vitrectomy probes, with selected macular diseases and non-complicated vitreous hemorrhages, was published by Oshima et al. [8]. Although the fluid dynamics and cutting efficiency of the 27-gauge probes were lower when compared to the 25-gauge system, important aspects with the new tested devices were no need to convert to larger gauge instruments during the procedure and achievement of self-sealing sclerotomies, with no postoperative hypotony or endophthalmitis, which were observed in some of the initial studies using 23-gauge and 25-gauge probes [8, 14–17].

Nowadays, the indications for 27-gauge surgery have expanded. More complex cases including diabetic retinopathy, rhegmatogenous retinal detachment and nucleus fragments removal have been managed with the next generation of 27-gauge cutter probes [7, 9, 12–14, 18]. The thinner instrument can be safely introduced into smaller spaces between membranes and retina, serving as a multifunctional device and facilitating tissue dissection [7, 14, 18]. The decreased stiffness is still noted when compared to other gauges (i.e., half of the 25+ gauge stiffness), but the rigidity has been improved by shorter length needles or the introduction of a stiffness sleeve, such as the one released by Alcon [7]. Khan et al. published the largest case series using the 27-gauge vitrectomy system in 2016. This multicenter study enrolled 95 eyes that underwent 27-gauge vitrectomy surgery, using the Constellation Vitrectomy 27+ Total Plus Pak (Alcon, TX, USA) probe for a variety of conditions. No intraoperative complications and no conversions to larger gauge instrumentation were required. Overall, the visual acuity improved from the baseline and the surgeons mentioned no complaints on instrument rigidity.

**Fig. 1:**
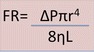
Flow rate of a pure/aqueous fluid according to the “Poiseuille’s law”; ∆P is the pressure difference across the length of the probe needle, r is the inner radius of the vitrectomy probe, η is the viscosity of the fluid and L corresponds to the length of the vitrectomy tube

Of particular importance, as per Poiseuille’s law (Fig. 1), decreasing the inner lumen of the vitrectomy probe resulted in more resistance to flow and diminished the overall flow rate. The decreased vitreous flow rate or so-called “cutting efficiency” observed with those smaller 27-gauge instruments was at least partially overcome by the development of higher cutting speed devices and high aspiration levels along with duty cycle improvements (described in next sections) that are already present on some of the commercially available platforms [7, 14, 19, 20]. In a recent publication, the mean operative time was similar to the initial reports using the 23-gauge instruments, regardless the potentially reduced flow rate [12].

### Cutter speed

A substantial increase in cutter speeds has occurred since Machemer introduced the vitreous infusion suction cutter (VISC), in the 1970s. Current vitreous cutters are capable of delivering cut rates of up to 16,000 cpm (cuts per minute), depending on the vitrectomy platform (e.g., EVA; DORC International) [21, 22]. Although other components are involved in the vitreous flow rate (e.g., duty cycle), faster cutting speeds are generally associated with increased vitreous removal and therefore, surgery efficiency and shorter procedure time [20, 23]. The vitreous has an unpredictable flow behavior, difficult to characterize, due to its semi solid structure, composed of water, collagen fiber and hyaluronic acid [24] and differently from the balanced salt solution (BSS), that is easily aspirated, the vitreous requires cutting before going through the probe [25]. Therefore, a high cutting rate is desirable. The chopped vitreous has a lower viscosity than intact gel and is more easily aspirated even in reduced diameter instruments [24]. In addition, for the same flow rate, the higher the cut rate, the smaller the amount of vitreous (“bite size”) aspirated into the cutter, reducing both vitreous and retinal traction [19, 26]. Teixeira et al, assessing different 20, 23 and 25-gauge vitrectomy probes under porcine vitreous were able to demonstrate a decrease in retinal traction for every 500 cpm increase in the cut speed [26]. Rizzo et al. have also shown a lower rate of iatrogenical retinal breaks when performing surgery with a high speed vitrectomy system (5000 cpm) when compared to a lower cut speed machine [23]. Recently, Pavlidis et al., assessing a two-dimensional cutter probe (see next section), capable of cutting rates equivalent to 16,000 cpm in the DORC EVA (DORC International) platform, suggested that the higher cutting speed helped to ensure a faster vitreous removal when the performance was compared to a standard single port cutter of the same gauge [27]. The liquefaction and excision of the vitreous body using an ultrasound, is another concept under investigation, with a prototype developed by Bausch and Lomb (Baush and Lomb, St. Louis, MO, USA). Initial reports under porcine eyes have shown promising results using 23 and 25-gauge probes, with no macroscopic retinal defects and no compromise of the BSS and vitreous flow rate when using different ultrasound powers, regardless of the gauge size [22, 28, 29].

### Cutter port and blade design

The vitreous cutter port geometry and blade design may have a great influence in vitrectomy surgery from the fluidics and safety standpoints [30]. DeBoer et al. demonstrated that increasing the port diameter resulted in higher flow rates (both in water and porcine vitreous), but only to a certain limit. When the port diameter became larger than the internal lumen of the vitrectomy cutter, less effect was noted on the overall flow rate [31]. In the same study, when assessing different vitrectomy tips, a grater model demonstrated to be a safer option for vitreous shaving, avoiding direct cut of the retina. The authors stated that designing port geometries with the appropriate port size might allow a combination of maximal flow and accurate cutting.

The standard guillotine-shaped vitrectomy blade has been used for many years. However, its movement to cut the vitreous, with complete port closure, may result in flow instability, fluid acceleration and retinal traction [30, 32]. New blades have been designed in order to increase cut rate and overall surgery efficiency, while maintaining a safe environment.

Rizzo assessed a modified 23-gauge probe, with a “hole” in the standard guillotine blade, showing increased flow and cut rates, which could be associated with less retinal traction [33]. However, using a similar blade design under egg albumen to simulate vitreous, Rossi et al. demonstrated higher particle acceleration than regular blades, which may lead to dangerous retinal movement [30].

**Fig. 2:**
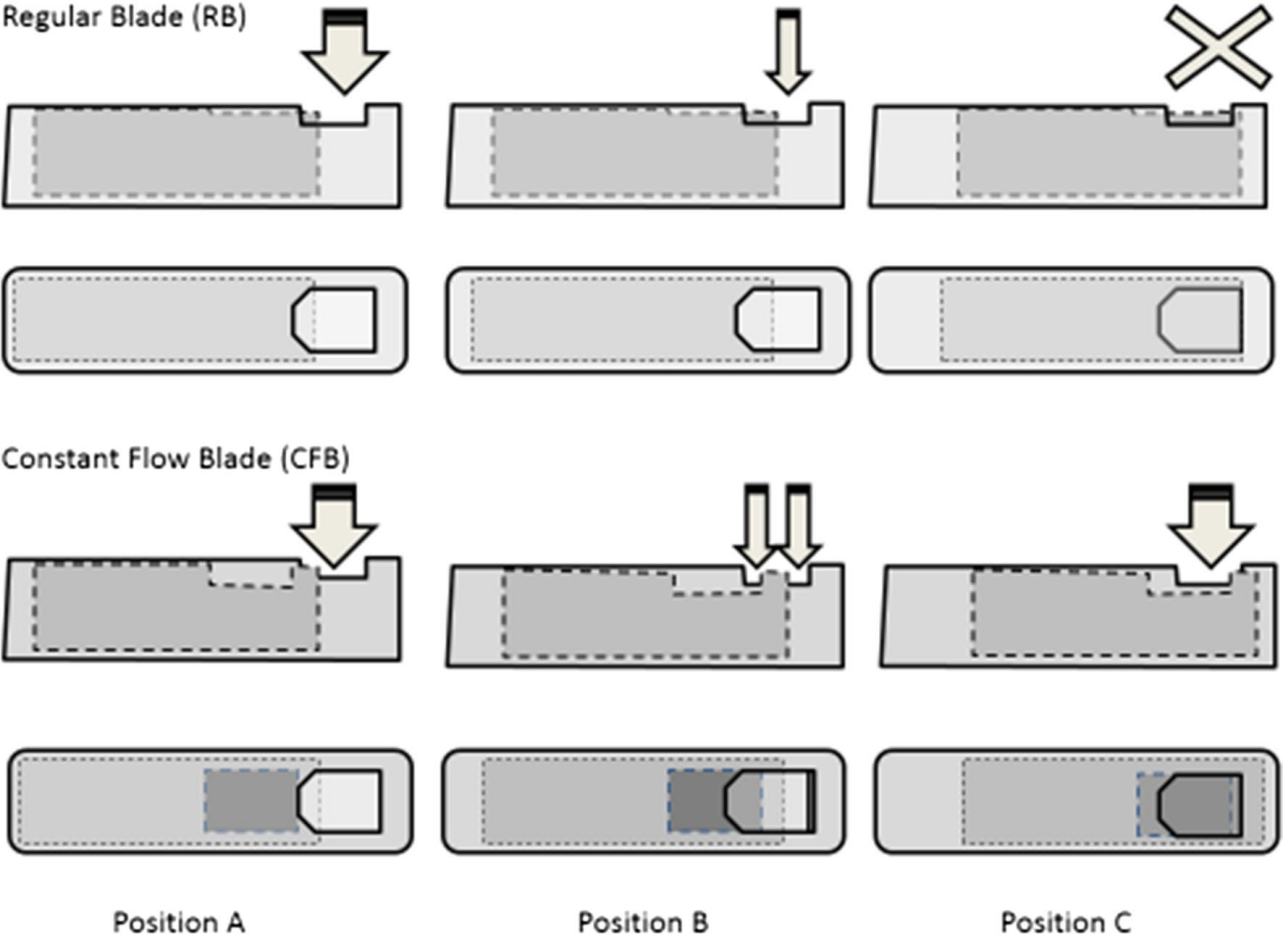
Comparison of the regular guillotine-shaped and the constant flow blade (CFB). The regular guillotine-shaped blade (*upper portion*) completely closes on position C, whereas the CFB maintains the same amount of port opening during the total cutting cycle, while chopping the vitreous at the proximal and distal edge of the blade (position C—lower portion). Image courtesy and reproduced with permission of Dr. Tomasso Rossi. First available on Rossi et al. [32]

A new port and blade shape, known as Constant Flow Blade (CFB; Twedge Cutter Blade; Optikon 2000 Inc, Rome, Italy), was also evaluated by Rossi et al. [32]. The device maintains the amount of open port surface constant all over the cutting cycle, and cuts both at the proximal and at the distal end (Fig. 2), in a concept that was initially patented by Hayafuji et al., back in 1992 [34]. The authors compared the performances of 23-gauge probes with a regular guillotine blade and a CFB. The duty cycle of the CFB showed trends to 100% (independent of the cut rate), where as the regular blade decreased as cut rate increased. The BSS flow rate of the CFB was independent of the cut rate and was superior to the regular blade regardless of aspiration settings and pump type. When using egg albumen to simulate vitreous conditions, the CFB showed an improvement of the flow rate up to 1000 cpm, after which it remained constant. The regular blade also showed a rise of the flow rate, as cut rate increased, but at much lower levels than the CFB. Kinetic energy fluctuation was more pronounced when using the regular blade, which could be translated into higher fluid acceleration and more retinal traction. In the same study, 12 cases were performed with the CFB and the authors suggested that the experimental efficiency could also be felt surgically, although details on the patients outcomes were not shown.

Similarly, Claus Eckardt and Mitrofani Pavlidis, in conjunction with DORC, have developed a double-port two-dimensional cutter (TDC—Additional file 1: Video 1; available in 23, 25 and 27-gauge), which features a larger rectangular aperture in the inner lumen, with two sharp cutting edges, cutting vitreous in a forward and backward movement during each cycle, reaching rates of up to 16,000 cpm [14, 21, 27, 35]. No matter the position of the blade, the port is never occluded, leading to a duty cycle of almost 92%, with constant aspiration flow, even at higher cut rates [21]. Osawa et al. carried out an experiment comparing the 27-gauge TDC and a standard 27-gauge probe, under BSS and porcine vitreous. The 27-gauge TDC flow rate under porcine vitreous was about 50% higher than the standard one. The BSS flow rate, remained constant regardless of the cut rate [14].

### Cutter technology, duty cycle and their fluidics interaction

**Fig. 3:**
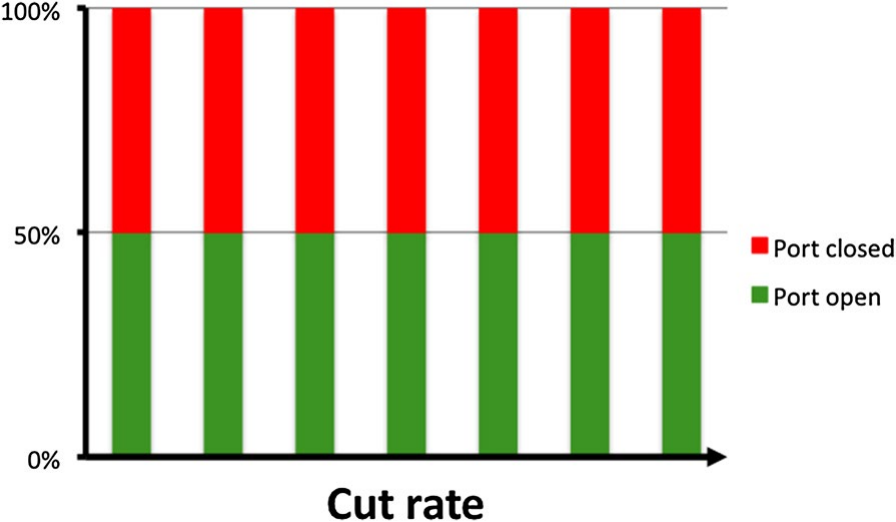
Duty cycle of a typical eletric cutter, with a 50% value (the port is open approximately 50% of the whole cutting cycle) up to the maximum cut speed set up on the machine

**Fig. 4:**
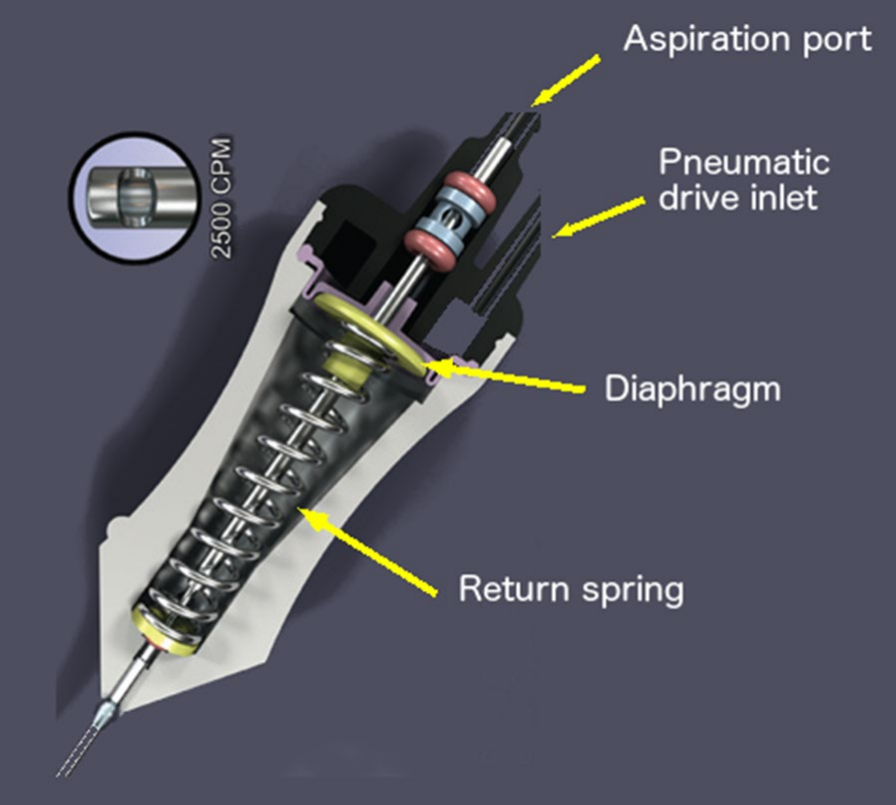
Pneumatic spring return driven vitrectomy probe. An air pulse pushes down the diaphragm located inside the vitrectomy probe, leading the port to a closed position (the guillotine movement); at the same time, a spring is compressed and forces the diaphragm back to the open port position. Image provided by Alcon, USA

Duty cycle (DC) is the percentage of open port time for each complete cut cycle (DC = open port time/time of a complete cut cycle) and has a major impact on fluidics during vitrectomy surgery. The initial electric cutters had a fixed 50% DC (the port was open approximately 50% of the whole cutting cycle), maintaining a near constant flow rate, up to the maximum cut speed set up on the machine (Fig. 3) [19]. Differently, the original pneumatic driven cutter relied on a spring return mechanism in which an air pulse pushes down the diaphragm located inside the vitrectomy probe, leading the port to a closed position (the guillotine movement); at the same time, a spring is compressed and forces the diaphragm back to the open port position (Fig. 4).

**Fig. 5:**
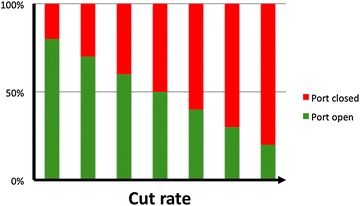
Time of port open and port closed of a typical pneumatic spring return cutter. As cut speed increases the duty cycle decreases to some degree

This spring return mechanism, however, limits the control over the duty cycle: as cut speed increases the DC decreases or in other words, the amount of time the port remain open decreases along with a disproportionately lower flow rate (Fig. 5) [19, 25]. Ribeiro et al. assessed the water and vitreous flow rates and DC of different ultra-high speed spring return pneumatic cutters (20, 23 and 25-gauge) at variable aspirations levels. The DC reduced as speed increased for all gauges [36].

**Fig. 6:**
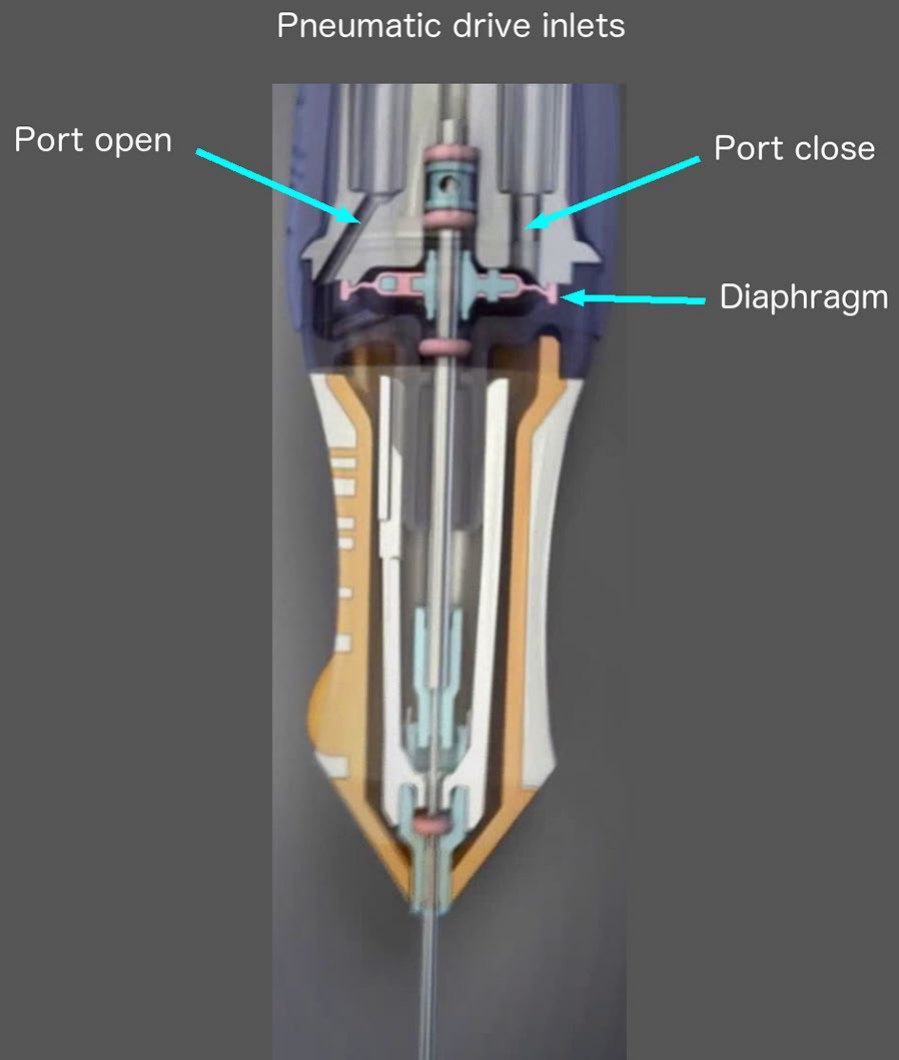
Dual pneumatic vitrectomy cutter. An air pulse pushes down the diaphragm located inside the vitrectomy probe, leading the port to a closed position (the guillotine movement); another air pulse, in a separate air line, pushes the diaphragm back to the port open position. Image courtesy of Alcon (Ultravit^®^ probe—Alcon, USA)

**Fig. 7:**
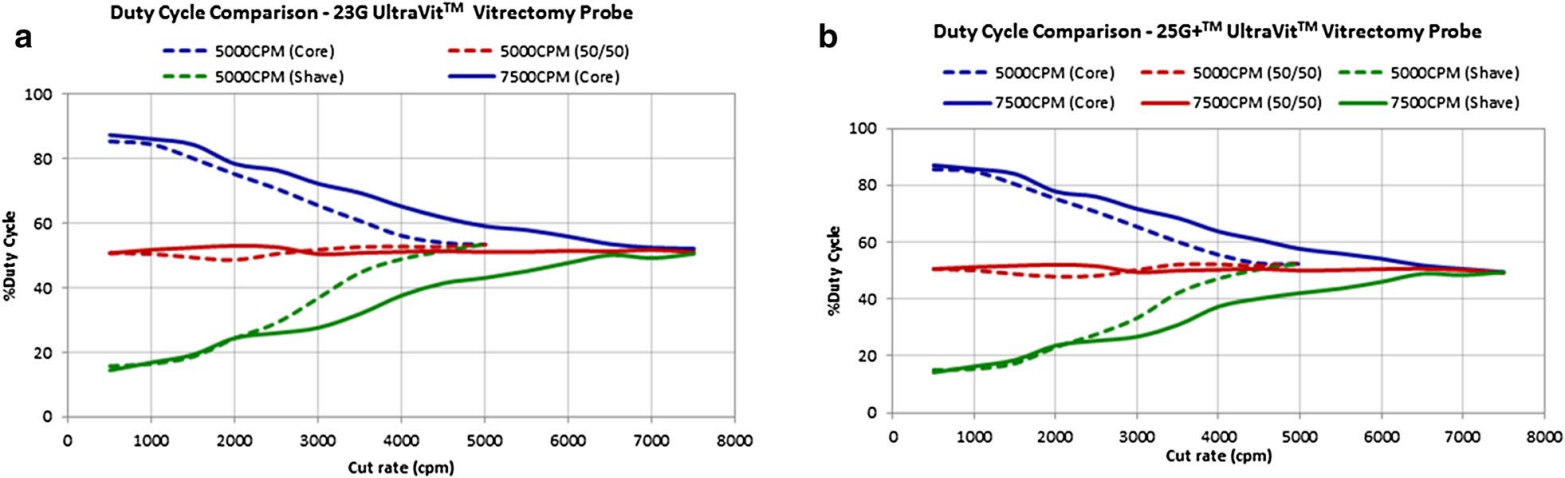
Duty cycle (DC) pattern of 23 gauge (**a**) and 25+ gauge (**b**) dual pneumatic cutters according to the cut rate. As cut rate increases there is a trend to a 50% DC, regardless of the initial selected mode (50/50, shave mode or core mode). *Source*: Alcon data on file/Test Report 954-2020-003

Instead of using a spring to return the guillotine to the original position, the dual pneumatic probes use separate air lines to both open and close the vitrectomy port (Fig. 6; Additional file 2: video 2). This allows the DC to be controlled independently of the cut rate [19, 24, 37–39] with customized modes: “port biased open” or core mode (the port remains open for the majority of time), 50/50 mode (the port is open 50% of the time) and “biased closed” or shave mode (the port remains closed for the majority of time). Therefore, besides the cut rate and vacuum/aspiration levels, the surgeon can count on another variable in order to control the flow rate. However, even when using controlled modes, there is a trend to a 50% DC as cut speed increases (Fig. 7) [24, 39]. Diniz et al. assessed the performance of dual pneumatic probes of different diameters (20, 23 and 25-gauge), using a high-speed system (up to 5000 cpm), in numerous aspirations levels, with a biased open DC, under water and porcine vitreous conditions. The author showed that DC decreased, converging to a value close to 50%, as cut rates increased. The water flow rate followed the DC pattern, decreasing as cut rate increased. The vitreous flow rate increased as the cut rate increased [38].

In general terms, in the core mode, when increasing the cut rate, the only time segment that can be reduced in order to achieve a higher cutting speed is the open port time, and the DC reduces. In the same manner, to increase the cut rate in the shave mode, the only segment that can be changed to obtain a higher cutting speed is the time the port remain closed. Consequently, the DC increases [40]. The water flow rate tends to follow the DC pattern (i.e., if the DC decrease so does the water/BSS flow rate). The vitreous flow rate was reported with some different results in the literature. Diniz et al. have shown that under cutting speeds of up to 5000 cpm, the vitreous flow rate tends to increase even when the DC decreases. This could be explained by the vitreous fragmentation, resulting in less resistance to aspiration and improving the flow rate [24, 36, 38]. However, a new study from Abulon et al. assessing the vitreous behavior in porcine eyes during high-speed vitrectomy, with dual pneumatic cutters (23, 25 and 27-gauge), have demonstrated that vitreous flow rates at 7500-cpm, under biased open mode, was consistent with previously reported decrease in water and BSS flow rates with increasing cut rate. The authors stated that because resistance to flow is associated with increased vitreous viscosity, increased cut rate (which increases vitreous fragmentation and lowers vitreous resistance to flow) causes the fluid dynamics of vitreous to become similar to those of BSS (i.e., flow decreases with increased cut rate) although it still maintains efficient aspiration flow similar to 5000-cpm cutters. When using biased closed and 50/50 DC modes, the vitreous flow rate increased with higher cutting rates [41].

Despite the theoretical differences between the original spring return pneumatic cutters and the dual pneumatic driven cutters, Fernandes et al. recently compared the two mechanisms, under water and porcine vitreous, using two commercially available vitrectomy systems, in different probe sizes (20, 23, 25-gauge) and under different cutting speeds [42]. The dual pneumatic cutter had a modulated DC set to the biased open mode. Interestingly, the authors were able to show similar DCs, vitreous and flow rates, with only small differences between the two systems, reinforcing the idea that both driven mechanisms may have similar performances under high cutting rates.

**Fig. 8:**
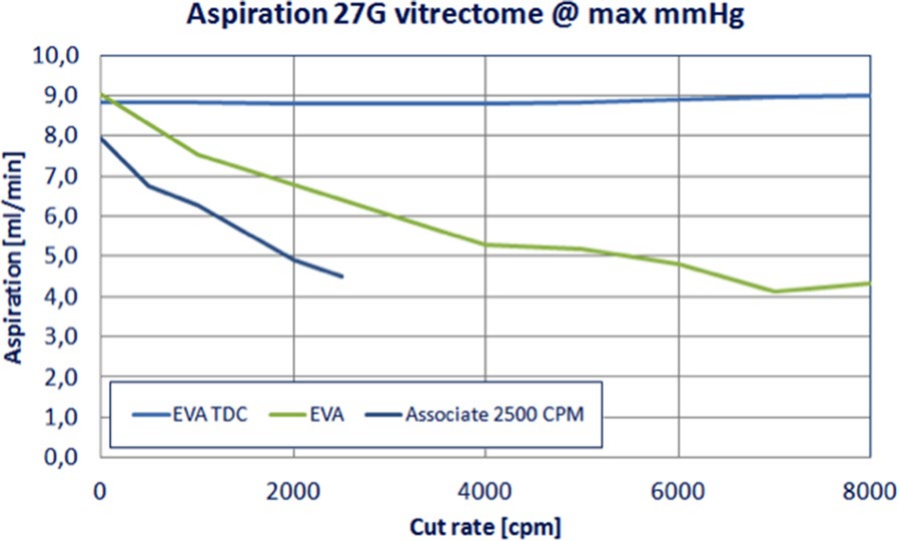
Basic saline solution (BSS) aspiration flow rate pattern of three different 27-gauge vitrectomy cutters according to the cut rate. The two-dimensional cutter (TDC; DORC International) shows consistency of the flow rate irrespective of the cut rate, illustrating the approximately 100% duty cycle mechanism of new vitrectomy cutter designs. Figure courtesy of DORC International

As previously mentioned, new commercially available port and blade designs, capable of a double cutting movement, may be associated with DC of approximately 100% (since the vitrectomy port is never completely closed), constant flow rates and shorter operation times (Fig. 8). Most important, the smother flow may lead to less acceleration and a tractionless environment, although safety studies are still necessary to confirm those findings (Additional file 3: video 3) [14, 21, 27, 32].

### Vitrectomy pumps

**Fig. 9:**
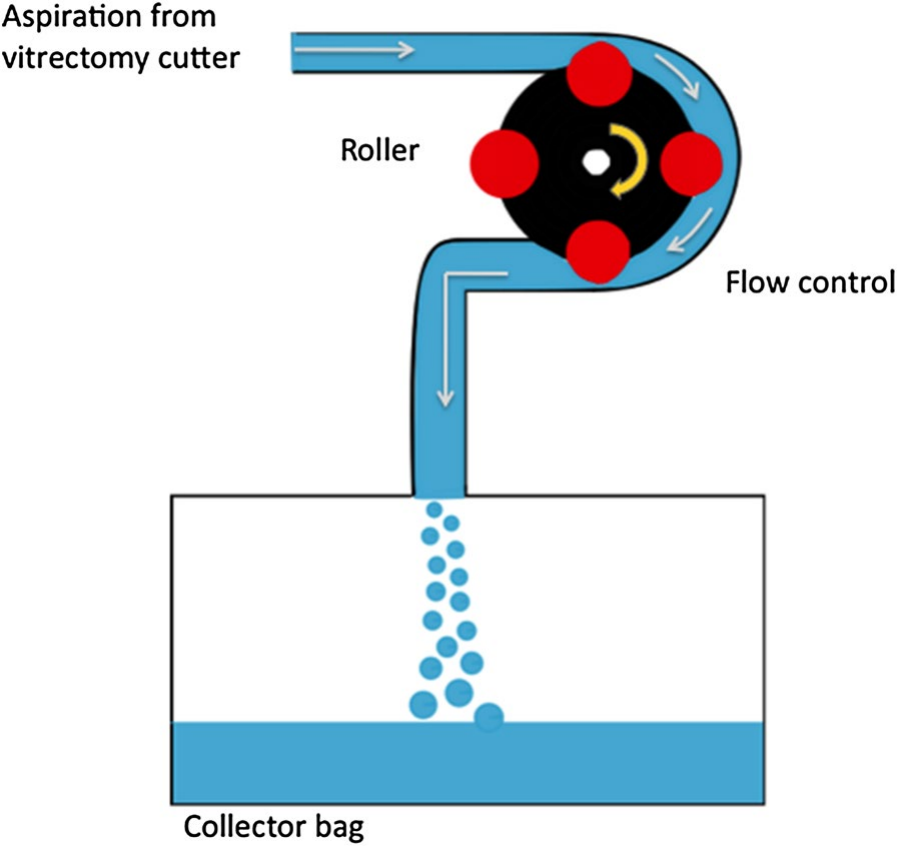
Peristaltic pump. The fluid within a tube is compressed and forced to dislocate by the roller. A gradient of pressure is created between the infusion and the point of pressure, leading to aspiration and directly controlling flow by the roller rotational speed

**Fig. 10:**
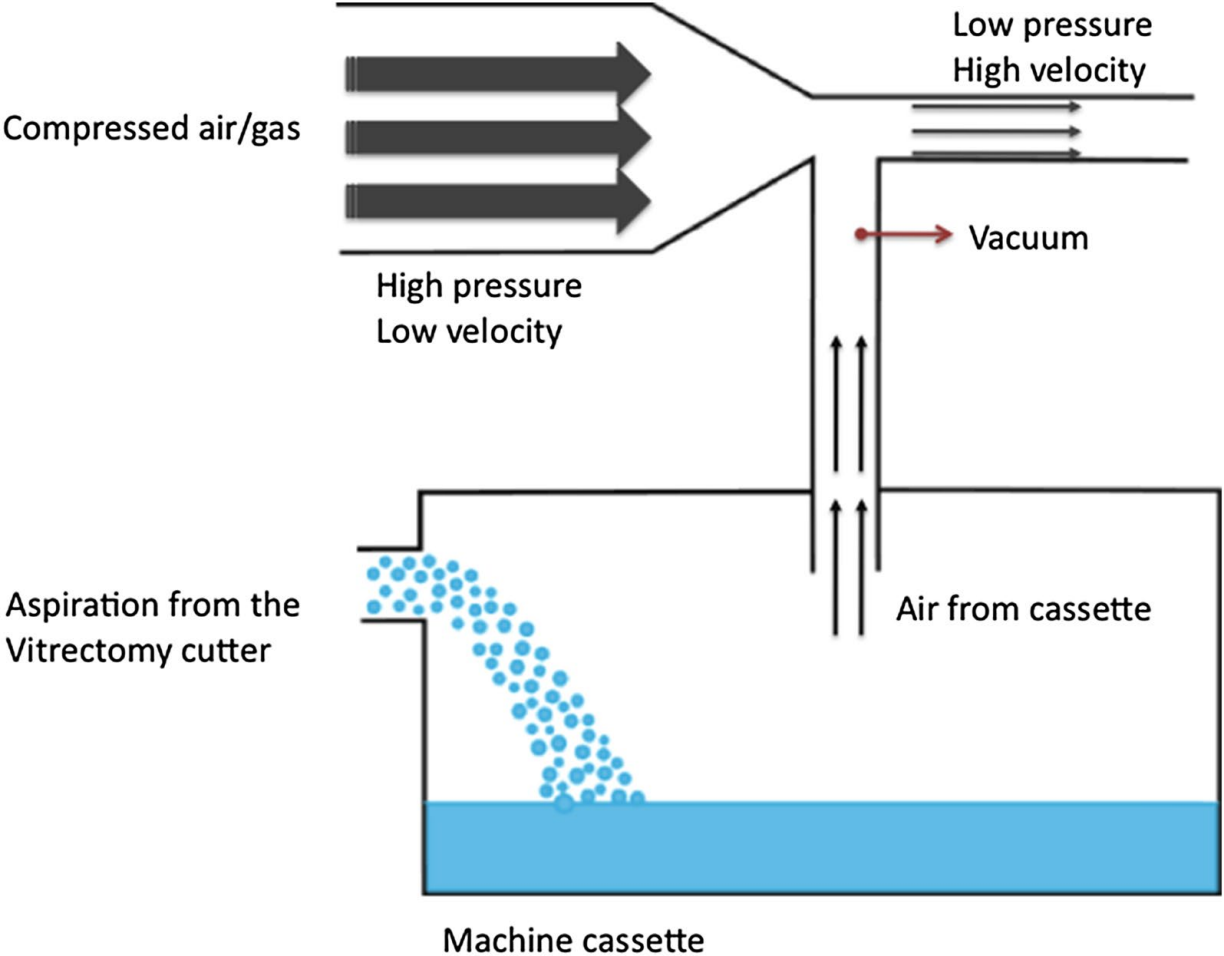
Venturi pump. Vacuum is generated by a flow of air/gas and is transmitted into the cassette. The air inside the cassette is aspirated and flow from the cutter aspiration line reaches the cassette, as a result of the created vacuum. The flow is controlled by the vacuum levels

Ophthalmic vitrectomy machines have typically incorporated a peristaltic pump, a venturi pump or a combination of both to manage fluidics during surgery. Peristaltic pumps work with rollers compressing and dislocating the fluid within a tube, creating a gradient of pressure between the infusion and the point of pressure, leading to aspiration and directly controlling flow (Fig. 9). Once an occlusion occurs, the vacuum will start to increase till a preset value in order to maintain the desired flow. Both flow and maximum vacuum points can be set on the machine prior to surgery. Drawbacks of peristaltics pumps include pulsatile vacuum, mild flow fluctuations as the roller compresses the tube and inability to proportionally control the vacuum in the presence of bubbles in the tubing system. Venturi pumps directly control vacuum to generate flow. The vacuum is created using the effect of same name, with air/gas flowing over an opening and reducing the pressure inside the ophthalmic cartridge (Fig. 10). The flow varies according to the strength of the vacuum and can only be estimated. A precise flow control is difficult to achieve, especially when changing the media (e.g., going from BSS to the vitreous) [19–21, 43, 44]. A study published by the European Vitreoretinal Society (EVRS) assessed primary vitrectomy for rhegmatogenous retinal detachment using either a peristaltic or venturi pump. The authors have shown that venturi pumps were associated with a significantly higher failure rate (the retina remained detached by the end of the study) when compared to peristaltics pump vitrectomy. The difference of failure rate between pumps was significant when comparing 20-gauge vitrectomy (not significant when comparing 23-gauge only vitrectomy). In addition, the study demonstrated that high speed vitrectomy decreased failure rate for venturi pumps [45]. Both observations may not be surprising, since a smaller gauge and a higher cutting rate are part of the “port-based flow limiting”, which reduces the amount of vitreous aspirated into the cutter for anyone cut and may decrease tractional complications [19, 20].

**Fig. 11:**
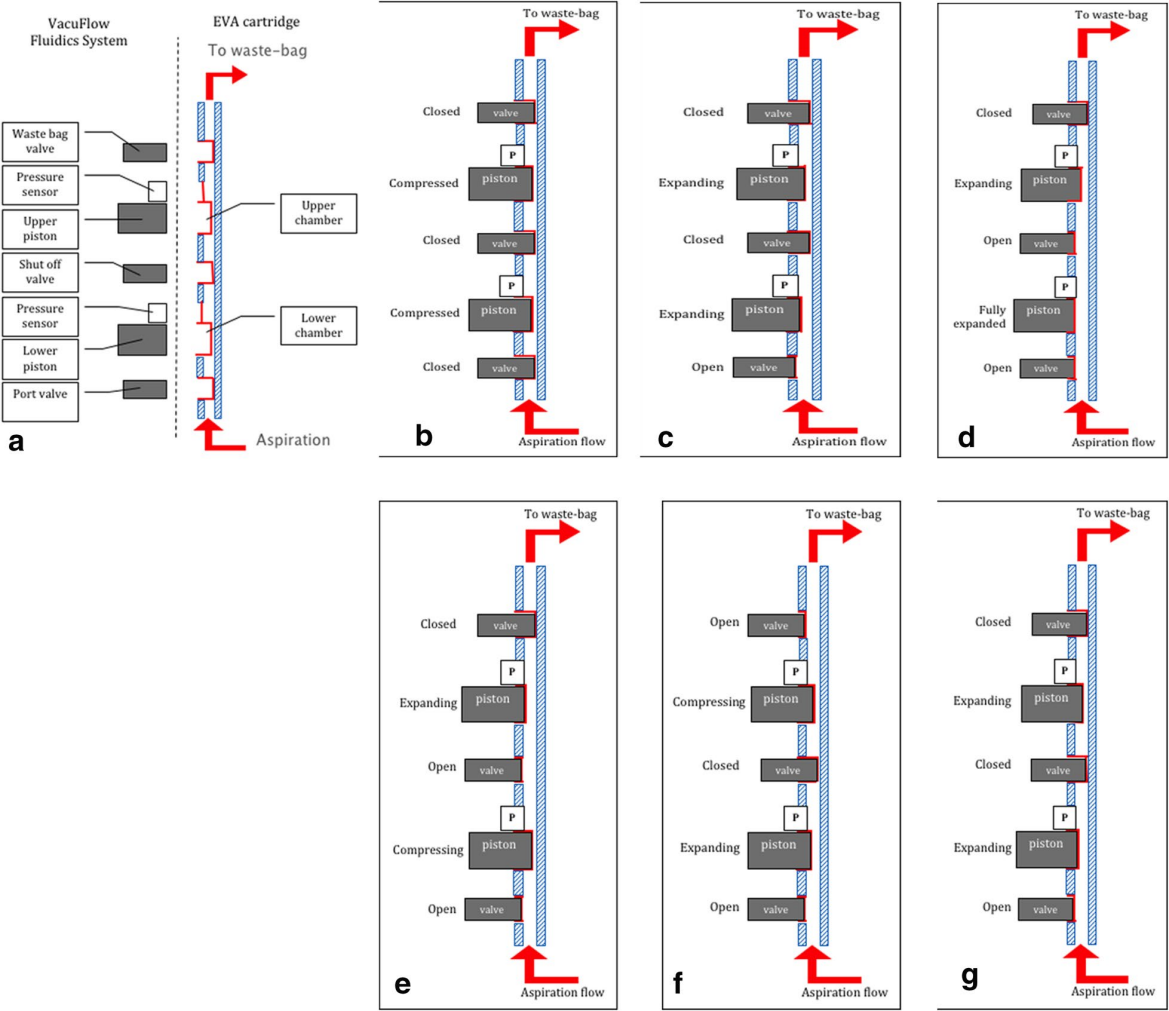
Vacuflow VTI (Valve Timing Intelligence). The EVA cartridge contains two small flow chambers (6 ml), which volumes are controlled by computer-based pistons, valves and high-sensitivity pressure sensors located on the EVA platform (**a**). Sequence: **b** At the start of the sequence both chambers of the cartridge are compressed and the valves are closed. **c** When there is a demand to generate aspiration flow the port valve opens and the lower chamber expands. Due to this expansion the fluid will be drawn into the fluid displacement chamber, resulting in aspiration flow. The speed of the expansion determines the amount of aspiration flow: higher speeds will achieve higher flow rates. The associated pressure is measured with the chamber pressure sensor. Meanwhile the fluidics system creates an identical pressure in the upper chamber by expanding the upper chamber and keeps this equal to the pressure of the bottom chamber. **d** As soon as the lower chamber is fully expanded the shut off valve opens. At this point the pressure in both chambers are identical, eliminating pressure pulsations in the aspiration line. **e** From this point the lower chamber is being compressed in order to empty the chamber, while the upper chamber is being further expanded. The expansion of the upper chamber is faster than the bottom chamber due to the fact that it must generate aspiration flow and displace the fluid from the lower chamber into the upper chamber. **f** Once the lower chamber is fully compressed the shut off valve closes and the lower chamber expands to generate the aspiration flow. Meanwhile the waste bag valve opens and the fluid in the top chamber is compressed emptying it into the waste bag. **g** As soon as the upper chamber is emptied into the waste bag it expands to create a pressure similar to the lower chamber. Once the lower chamber is fully expanded the cycle repeats. Figure courtesy of DORC International

In 2012, DORC introduced the Vacuflow Valve Timing Intelligence (VTi) technology, available on the DORC EVA platform, which is neither a peristaltic, nor a venturi based system, but is capable of providing both flow and vacuum mode. The EVA cartridge contains two small flow chambers (6 ml), which volumes are controlled by computer-based pistons, valves and high-sensitivity pressure sensors located on the EVA platform (Fig. 11), generating a fast vacuum response (vacuum set is achieved in 0.3 s) and flow control (0.1 ml accuracy), eliminating unwanted flow fluctuations [21, 35]. The use of vacuum mode for detaching the hyaloid and the flow mode while performing delicate peripheral vitrectomy, next to the retina, with rigid aspiration control, are examples of how this technology could further be applied to enhance surgery safety.

### Small gauge overall considerations

Small gauge instruments (23, 25 and 27-gauge) are the procedure of choice of most of the vitreoretinal surgeons. Shorter operation time, less postoperative inflammatory reaction and conjunctival scarring, fast postoperative recovery, potential self-sealing wounds and less vitreoretinal traction are among the main advantages over 20-gauge vitrectomy. The initial hypotony concerns when using 23 and 25-gauge instruments following sutureless surgery were overcome with angled or two-step techniques of wound construction, although complete self-sealing sclerotomies are still not achievable in every single case [7–11]. Regarding the rate of endophthalmitis in sutureless microincision surgery (23 and 25-gauge), a systematic review by Govetto et al. did not find an increased risk when compared to 20-gauge surgery, although the authors recommended caution when interpreting the results due to the small number of events reported [46].

Major drawbacks of 25-gauge vitrectomy, when it was first introduced in 2002, were decreased illumination and instrument stiffness. New light sources managed to “put some light in the dark” (see next section) and probe modifications enhanced the instrument rigidity, although, by its own nature, it continues to be more fragile than 23-gauge. The smaller diameter, with decreased flow rates when compared to 20 and 23-gauge probes, actually contribute to the port based flow limiting and combined to high cutting rates and duty cycle control, results in less pulsatile vitreoretinal traction and enhance safety, as proposed by Steve Charles [20, 47]. An entire line of vitrectomy accessories is available and the indications cover the whole spectrum of vitreoretinal pathology [48].

Twenty-three gauge vitrectomy introduced by Eckardt, in 2005, came to address some of those early issues with 25-gauge probes, mainly concerning the instrument flexibility, lower flow rates and decreased illumination. The 23-gauge instruments had a smaller diameter than 20-gauge and were more rigid than 25-gauge, providing better illumination and facilitating the access to the peripheral vitreous (by eye rotation), without having to worry about instrument bending. Initially adopted for macular procedures, 23-gauge now incorporates the whole range of vitreoretinal procedures and is the device of choice by the majority of surgeons around the world according to 2014 ASRS pat survey.

Twenty-seven-gauge sclerotomies, with a smaller diameter (0.4 mm for 27-gauge; 0.5 mm for 25-gauge; 0.6 mm for 23-gauge) can be made perpendicular to the sclera and no angled or two-step techniques are required. The benefits of less inflammatory reaction, fast wound closure, less vitreous incarceration and fast postoperative recovery have potentially improved. The flow rates, as expected, have reduced when compared to 23 and 25-gauge systems, but increased cutting speed, combined with duty cycle control and newer cutter/blade shapes (e.g., two-dimensional cutter, by DORC International) as previously mentioned, have brought it to acceptable rates. Also, the rigidity was enhanced by the introduction of reinforcement sleeves (e.g., stiffness sleeve by Alcon, USA). Much like during the introduction of 23 and 25-gauge, 27-gauge instruments were initially used for selected cases. The indications, however, have already expanded to more complex surgeries. The smaller vitrectomy diameter and the port aperture closer to the tip can be positioned between narrow spaces, allowing membrane dissection and serving as a multifunctional instrument. The concerns about lack of endoillumination (see next section) have been resolved by the introduction of powerful Xenon, LED and future laser light sources [7, 8, 12, 13, 18, 49].

## Endoillumination

Numerous advances in endoillumination have occurred in the last decade, including the release of more powerful light sources, the usage of light filters to enhance tissue visualization and safety and the integration of chandeliers into complicated cases to allow bimanual surgery.

**Fig. 12:**
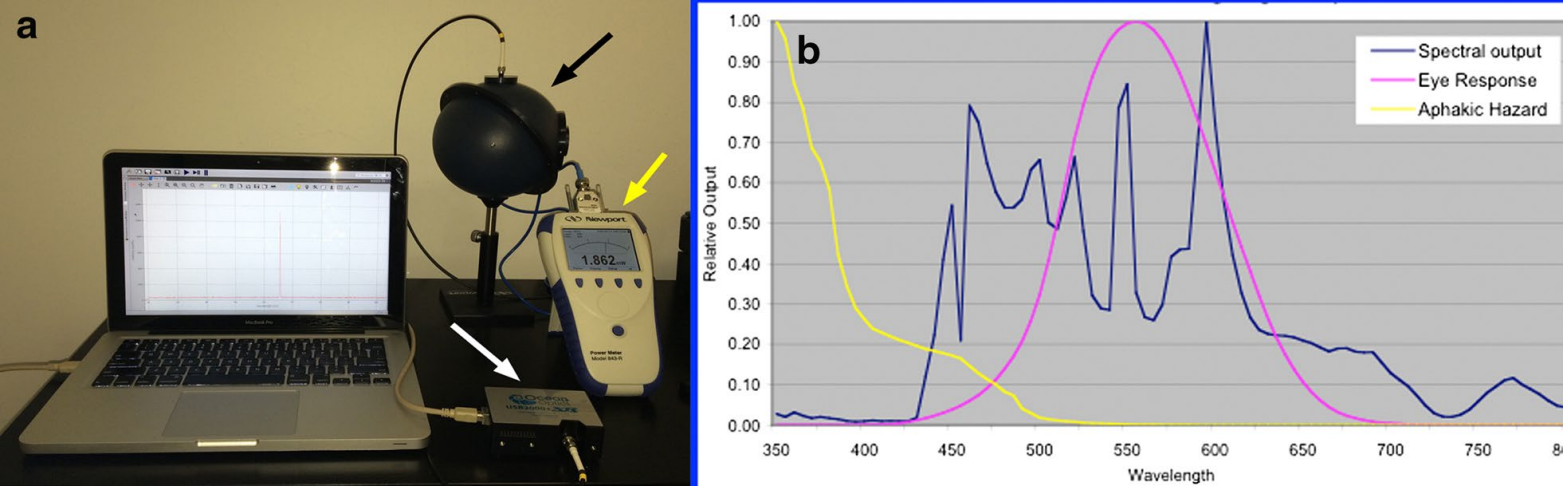
**a** Set up for obtaining the spectral curve and power output of a light source. The spectrophotometer (*white arrow*) and power meter (*yellow arrow*) are linked to the integration sphere (*black arrow*). The light shining into the integration sphere generates a spectral curve captured by the spectrophotometer also linked to the computer software. The power obtained is directly shown by the power meter. **b** Spectral curve of a light source (*blue curve*), as a function of wavelength and intensity output, against an aphakic hazard curve (*yellow*) and a photopic eye response curve (*purple*)

During vitrectomy surgery, retina surgeons need to be concerned about photochemical toxicity, as determined by Ham et al., in his study on *Rhesus* monkeys [50]. Essentially, as a result of his observations, an aphakic hazard curve was created showing that an increased risk of toxicity occurs with UV/blue light wavelengths exposure. Thus, when calculating the safety of a light source, after obtaining its specific spectral output curve (Fig. 12), the aphakic hazard sum (the standard measure of a light source safety) is achieved by the intersection of that light source spectral curve with the aphakic hazard curve [47, 50].

**Fig. 13:**
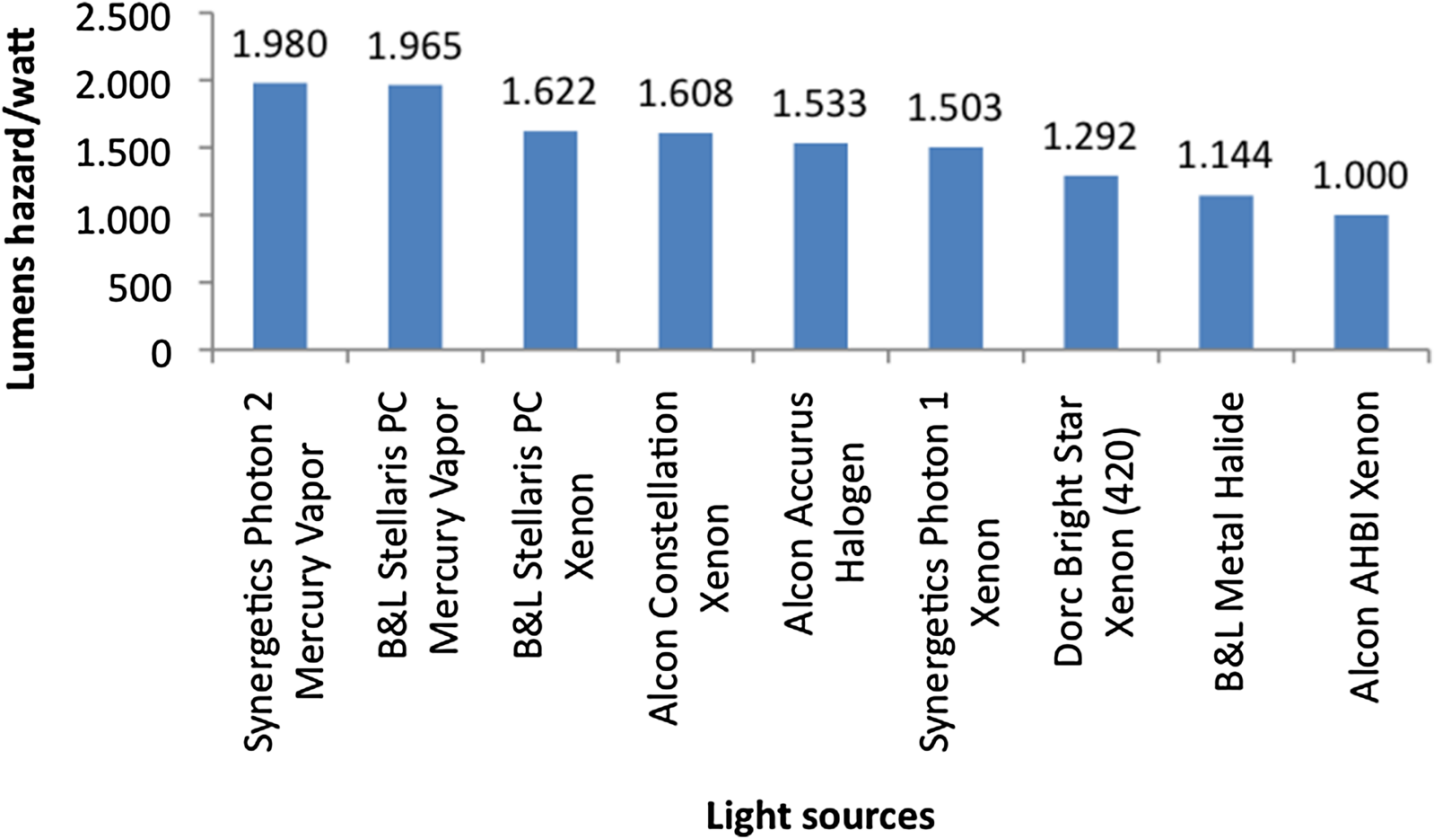
Safety calculations for different commercially available light sources, expressed in lumens hazard/watt (personal data). The higher the lumens necessary to create a watt of hazard, the safer the light source (for comparison, brightness, working distance and cone of illumination were all kept constant between the platforms)

The aphakic hazard sum can be inversed in order to express the number of lumens that are necessary to create a watt of hazard (lumens/hazard watt). The higher the lumens necessary to create a watt of hazard, the safer the light source (Fig. 13). Each light bulb has its own characteristic spectral curve and the only thing that can be modified by the manufacturer is the addition of a filter to the light source they have chosen.

**Fig. 14:**
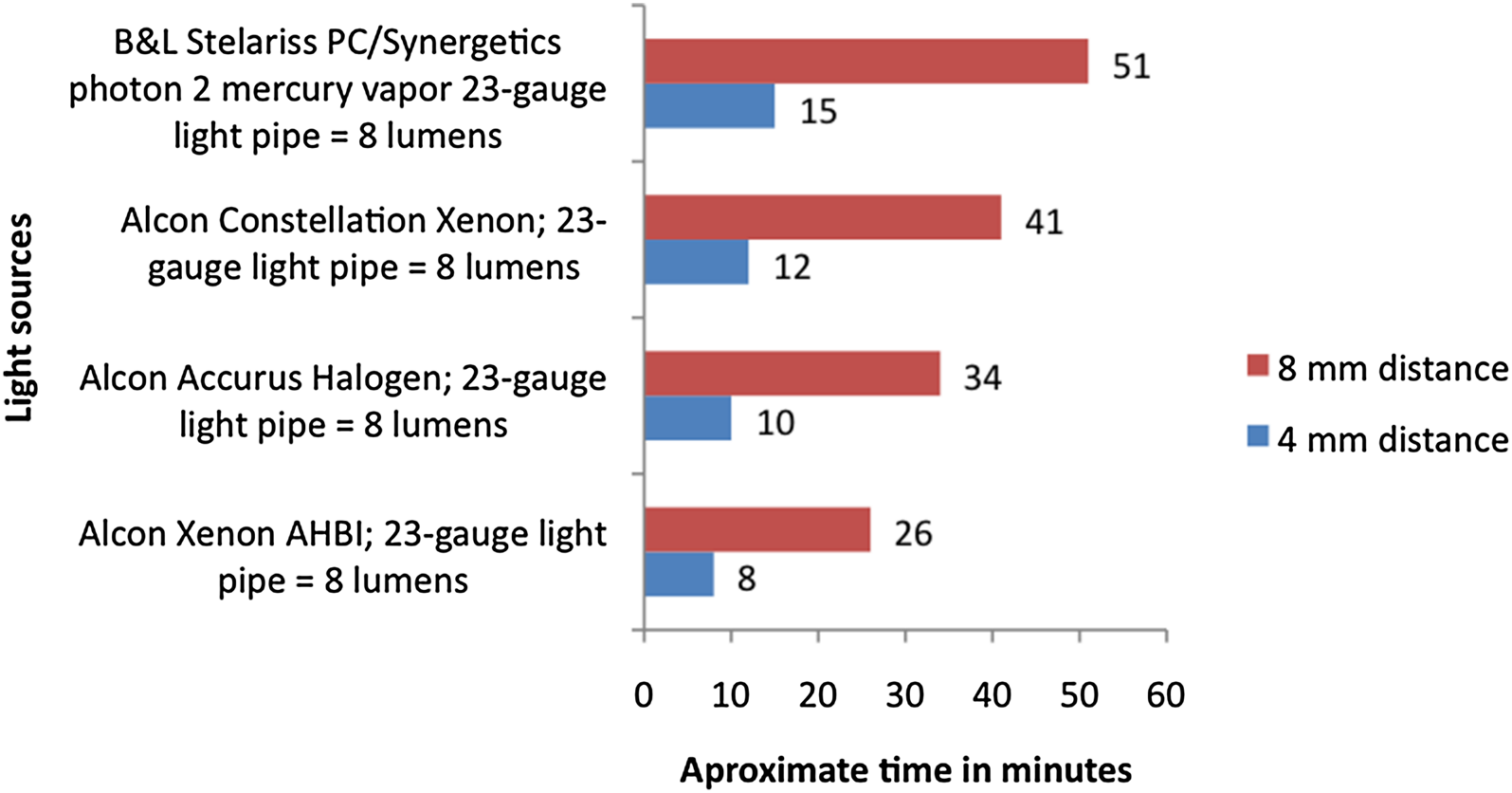
Retina threshold time of commercially available light sources according to their working distance from the retina (personal data)

Of more importance to surgeons however, is the retinal threshold time, which incorporates not just the inherent safety of the light source (aphakic hazard sum) but also the working distance, brightness, cone of illumination used (numerical aperture of the fibre) and the industry standard for toxicity of 25 J/cm^2^ [47]. This calculation allows surgeons to understand the theoretic time they can illuminate the retina under given settings and how changing those settings will impact safe working times. In evaluating all the variables in this equation it becomes quite clear that the biggest improvements in safety can be obtained by just increasing your working distance (Fig. 14).

Light filters can be used to improve safety and possibly enhance tissue visualization. Various light sources over the last 10 years have incorporated filters that can be used to exponentially increase the safety calculations of the light source. The Synergetics Photon, DORC EVA and B&L Stellaris PC all have incorporated some variant of a yellow filter to allow this improved safety. The filters have also been felt to possibly enhance tissue visualization. A multicenter study using the 3 filters (Amber, Green, Yellow) on the B&L Stellaris PC was performed a few years ago within which the surgeons were asked to grade the quality of the view obtained with each of the filters in place during different stages of a vitrectomy procedure. Although the baseline color of the light source was universally accepted as good for all parts of surgery some of the interesting surgeons preferences were: the preference of the Amber filter for Air Fluid exchanges (it was felt to reduce glare) and also for peeling the internal limiting membrane (ILM) when Brilliant Blue dye was used and the preference of the Green filter to remove the ILM with most other dyes [47].

The use of vital dyes may also be related to phototoxicity and damage to the neuroretina and to the retinal pigment epithelium (RPE). These type of substances, such as indocyanine green (ICG), tryplan blue (TB), brilliant blue (BB) and other commercially available dyes, are used to enhance tissue visualization (e.g., internal limiting membrane, epiretinal membrane and vitreous) during vitreoretinal surgery, in what has been known as chromovitrectomy [51–53]. However, after tissue staining, they may interact with light sources and induce photosensitizing effects at the retinal surface by an overlap of the emission spectrum of the light source and the absorption band of the vital dye used during the vitrectomy procedure. An increased number of free radicals would be released and could lead to retinal and RPE damage [54, 55]. Haritoglou et al, staining retinas from postmortem human donor eyes with 0.5% ICG were able to demonstrate damage to the inner retinal layers after illumination using the halogen light source, probably due to the overlap of the light emitted from the light device (between 380 and 760 nm) and the light absorbing-properties of ICG (maximum absorption beyond 600 nm and fewer absorption at lower wavelengths of 500 nm) [54]. Costa et al. also reported interesting findings when assessing the absorbance spectra of nine vital dyes (ICG, TB, BB, bromophenol blue, congo red, light green, fast green, indigo carmine and evans blue) diluted in three solvents (saline solution, glucose 5% and water) and their overlap with different light sources. In addition to the fact that the absorbance spectra varied with the solvent used, the authors have shown that the greatest overlap was found with integrated laser pathway (Photon Xenon; Synergetics Photon) and halogen lamp (Grieshaber GLS; GLS Corp.), and the least overlap was found with mercury vapor lamp (Photon 2; Synergetics). The lowest overlap values among the dyes were observed with ICG prepared in physiological saline solution, followed by indigo carmine, which showed low values for all three solvents compared with other dyes [55]. The surgeon must be aware that regardless of the substance chosen, as mentioned by Farah et al. [52], intravitreal injection of a vital dye poses a dose-dependent toxicity to the retinal tissue and the interaction with a light source may contribute to exert further retina damage.

Another important concept to understand in endoillumination is the brightness, which involves calculation of the output of a light source, using a power meter. The results are then modulated to our photopic response curve, to actually obtain the brightness perceived by us (expressed in lumens). The older halogen/metal halide light sources, when using a 20 gauge light probe had a power output of around 8 lumens. With the release of 25-gauge vitrectomy, many surgeons immediately complained about the lack of light. Testing revealed that the original light sources only had an output of 2–4 lumens in 25-gauge which was the initial impetus to the development of the stronger Xenon and Mercury Vapor light sources [47]. The stronger Xenon and Mercury Vapor light sources also allowed for clinically useful chandeliers and lighted instrumentation to be created, such as illuminated laser probes and vitrectomy picks, both contributing to a more assistant independent surgery, specially when working at the far periphery, where additional scleral depression by the assistant would usually be necessary.

Chandeliers, with multiple designs, have allowed “true” bimanual surgery and have a retinal threshold time in the order of hours, even when working at full output, which in summary, defines it’s significant safety. Additional file 4: Video 4 (courtesy of Dr. Oshima) shows an example of the 27-gauge Oshima Vivid Chandelier (Synergetics, USA). Although it has taken some time to integrate into clinical practice, recent data shows 75% of graduating retina fellows in North America will now regularly use a chandelier for complicated vitrectomies (unpublished data presented at the Retinal Fellows Forum, Chicago, 2014).

Recently, LED (light-emitting diode) light sources were introduced on the EVA platform (DORC, Netherlands) and the Versavit (Synergetics, USA). These LED bulbs offer the advantage of an extremely long life span of more than 10,000 h and allow surgeons the ability to titrate the color of their light source. There is also a laser light source which will be shortly released into the market (Katalyst Surgical, USA) incorporating 3 laser light sources which can be tuned to change the color of the light and provide a power capability on another level from the previous Xenon, Mercury Vapor and LED light sources. This increase in power will allow the usage of very small optical fibers, which can then be incorporated into our even smaller gauge instrumentation (25-gauge/27-gauge) to provide lighted 27-gauge instruments.

## Wide-angle viewing systems

A clear and wide view is essential during vitreoretinal surgery. This was made possible with the wide-angle viewing systems (WAVs) initially introduced in the 1980s [56–58] and which are continuously under development. The WAVs allow a panoramic view of the retina based on the indirect ophthalmoscopic principle. A lens gives an inverted image, which is then reinverted by a prismatic device, generally connected to the microscope. Access to the peripheral vitreous is provided, even in the presence of small pupils and corneal opacity, improving both surgical efficiency and safety. There are two main types of WAVs: contact lens and non-contact lens [59–65].

### Contact lens WAVs

The contact lens WAVs provide a better image resolution, contrast and stereopsis than non-contact systems. Once it is directly attached to the cornea, it eliminates natural corneal aberrations and limits the number of reflective surfaces [63, 64]. The lens is fixed by a ring or is held in place by a skilled assistant. Some currently available models come with self-stabilizing footplates. Although minimized, it may still require support from an assistant during complex peripheral vitreoretinal cases [63, 66, 67], which accounts for one of its major drawbacks. The field of view and magnification vary among the different models and manufactures (Table 1).

**Table 1:** Wide field contact lenses

	Manufacturer	Magnification	Field of view (°)	
			Static	Dynamic
MiniQuad	Volk Optical	0.48×	106	127
MiniQuad XL	Volk Optical	0.39×	112	134
HRX	Volk Optical	0.43×	130	150
Landers WF	Ocular	0.38×	130	146
Single use surgical WF	SMT	0.42×	NA	155
A.V.I lens	AVI	0.48×	130	NA

*A.V.I* Advanced Visual Instruments, *WF* wide field, *SMT* Sensor Medical Technology, *NA* data not available

### Non-contact lens WAVs

In the non-contact WAVs the lens is preplaced next to the cornea (which gives an inverted image) and needs an internal (e.g., Peyman–Wessels–Landers; Ocular Instruments, Bellevue, CA) or a separate prism system (e.g., Binocular Indirect Ophthalmo Microscope—BIOM; Oculus) to reinvert the image. The surgeon can adjust the field of view by changing the distance between the preplaced lens and the corneal surface [56]. The system doesn’t require an assistant to hold the lens in place. The cornea, however, must be covered by a viscoelastic material or be constantly irrigated to avoid dehydration and decreased fundus visibility. Preplaced lens condensation is another inconvenience during the procedure. Appropriate draping next to the eye should be performed to prevent that issue [63]. Although the field of view provided by the manufacturer (Table 2) gives the surgeon an idea of the system capability, it may vary under different surgical conditions: a dilated pupil, aphakic and air-filled eye may give the wider field of view [56].

**Table 2:** Non-contact wide field viewing systems

System	Manufacturer	Approximate maximum field of view (°)
BIOM (HD disposable lens)	Oculus	130
OFFSIS 120 D	Topcon	130
Merlin wide angle lens	Volk Optical	120
Resight 128 D lens	Carl Zeiss	120
PWL 132 D lens	Ocular	130
EIBOS 2 (132D) Moller-Wedel	Haag-Streit	124

*BIOM* binocular indirect ophthalmoscopy microscopy, *OFFSIS* optic fiber free intravitreal surgery system, *PWL* Peyman–Wessels–Landers upright vitrectomy lens

The combination of a contact and a non-contact wide field system may also offer advantages. Chihara et al. designed a prototype contact lens, with zero power, used in combination with a non-contact wide angle system and showed it not only prevented the cornea from becoming dry, but led to a smooth corneal surface, providing a good quality of view [59]. Some other studies assessed the simultaneous use of a magnifying contact lens and a non-contact WAVs: a wider field of view, along with no corneal dehydration and the potential ability to rapidly switch to a magnified macular view were observed [60, 61].

The non-contact WAVs have also been used along with scleral buckle procedures for the treatment of rhegmatogenous retinal detachment, under chandelier or slit lamp type endoillumination. Some possible advantages over the regular indirect ophthalmoscopy were mentioned: the image was not inverted, easier access to retinal breaks with dynamic scleral depression, even in small pupil eyes, and the ability of sharing the procedure image with medical staff and students. Pointed drawbacks were the risk of endophthalmitis, touching the lens with the illumination probe and vitreous wick from the scleral incision [62, 68, 69].

## Conclusions

Vitreoretinal surgery is a constant changing field. The advances in cutter technology, endoillumination and WAVs over the years were noticeable and the efforts in the development of new instruments most lead to a better surgical performance while increasing safety. New studies on endoillumination are being conducted and will soon show the latest safety patterns of light sources from different commercially available devices. More studies comparing the newest surgical blades, 27-gauge probes and the regular 23-gauge and 25+ gauge systems are necessary to allow consistent conclusions. As technology improves, the next WAVs will certainly enhance our ability to access peripheral vitreoretinal pathology while providing high definition images during surgical procedures.

## Additional files

**Additional file 1: Video 1.** The two-dimensional cutter. The vitreous is cut in a back and forth movement, doubling the cut rate in a single complete cut cycle. Video provided by DORC International.

**Additional file 2: Video 2.** Dual pneumatic vitrectomy cutter. An air pulse pushes down the diaphragm located inside the vitrectomy probe, leading the port to a closed position (the guillotine movement); another air pulse, in a separate air line, pushes the diaphragm back to the port open position. Video courtesy of Alcon (Ultravit^®^ probe—Alcon, USA).

**Additional file 3: Video 3.** Regular/guillotine blade (*left*) and the two-dimensional cutter (*right*) under porcine vitreous conditions filmed with a high-speed camera. The flow when using the two-dimensional cutter seems smoother compared to the regular guillotine-blade, with a duty cycle of approximately 92%. The first one eventually causes turbulence and repulse of the gel when the vitrectomy port is closed. Video by DORC International.

**Additional file 4: Video 4.** 27-gauge Oshima Vivid Chandelier (Synergetics, USA). Video courtesy of Dr. Oshima.

## Abbreviations

TSV: transconjunctival sutureless vitrectomy
VISC: vitreous infusion suction cutter
BSS: balanced salt solution
CFB: constant flow blade
TDC: two-dimensional cutter
DC: duty-cycle
ICG: indocyanine green
TB: trypan blue
BB: brilliant blue
ILM: internal limiting membrane
WAVs: wide-angle viewing systems
WF: wide field
AVI: Advanced Visual Instruments
SMT: Sensor Medical Technology
NA: not available
BIOM: binocular indirect ophthalmoscopy microscopy
OFFSIS: optic fiber free intravitreal surgery system
PWL: Peyman–Wessels–Landers
